# CRISPR-Based Gene Therapies: From Preclinical to Clinical Treatments

**DOI:** 10.3390/cells13100800

**Published:** 2024-05-08

**Authors:** Marine Laurent, Marine Geoffroy, Giulia Pavani, Simon Guiraud

**Affiliations:** 1 INTEGRARE, UMR_S951, Genethon, Inserm, Univ Evry, Université Paris-Saclay, 91190 Evry, France; 2 SQY Therapeutics, 78180 Montigny-le-Bretonneux, France; 3 Children’s Hospital of Philadelphia, Philadelphia, PA 19104, USA

**Keywords:** CRISPR/Cas9, gene editing, blood disorders, neuromuscular disorders, β-hemoglobinopathies, Duchenne muscular dystrophy

## Abstract

In recent years, clustered regularly interspaced short palindromic repeats (CRISPRs) and CRISPR-associated (Cas) protein have emerged as a revolutionary gene editing tool to treat inherited disorders affecting different organ systems, such as blood and muscles. Both hematological and neuromuscular genetic disorders benefit from genome editing approaches but face different challenges in their clinical translation. The ability of CRISPR/Cas9 technologies to modify hematopoietic stem cells ex vivo has greatly accelerated the development of genetic therapies for blood disorders. In the last decade, many clinical trials were initiated and are now delivering encouraging results. The recent FDA approval of Casgevy, the first CRISPR/Cas9-based drug for severe sickle cell disease and transfusion-dependent β-thalassemia, represents a significant milestone in the field and highlights the great potential of this technology. Similar preclinical efforts are currently expanding CRISPR therapies to other hematologic disorders such as primary immunodeficiencies. In the neuromuscular field, the versatility of CRISPR/Cas9 has been instrumental for the generation of new cellular and animal models of Duchenne muscular dystrophy (DMD), offering innovative platforms to speed up preclinical development of therapeutic solutions. Several corrective interventions have been proposed to genetically restore dystrophin production using the CRISPR toolbox and have demonstrated promising results in different DMD animal models. Although these advances represent a significant step forward to the clinical translation of CRISPR/Cas9 therapies to DMD, there are still many hurdles to overcome, such as in vivo delivery methods associated with high viral vector doses, together with safety and immunological concerns. Collectively, the results obtained in the hematological and neuromuscular fields emphasize the transformative impact of CRISPR/Cas9 for patients affected by these debilitating conditions. As each field suffers from different and specific challenges, the clinical translation of CRISPR therapies may progress differentially depending on the genetic disorder. Ongoing investigations and clinical trials will address risks and limitations of these therapies, including long-term efficacy, potential genotoxicity, and adverse immune reactions. This review provides insights into the diverse applications of CRISPR-based technologies in both preclinical and clinical settings for monogenic blood disorders and muscular dystrophy and compare advances in both fields while highlighting current trends, difficulties, and challenges to overcome.

## 1. Introduction

**Figure 1:**
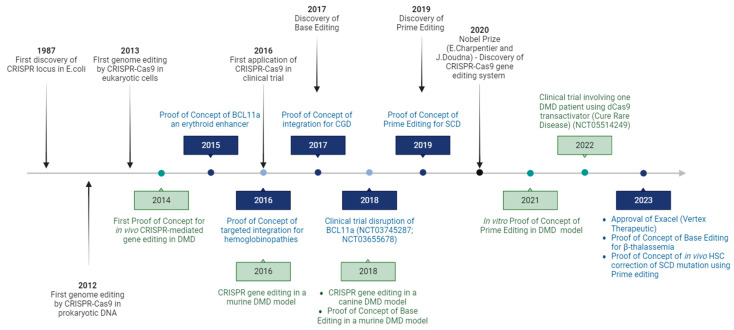
Timeline highlighting the important milestones in CRISPR gene editing field (black arrows). Achievements in CRISPR-based blood disease research are specified in blue and advents in CRISPR-mediated Duchenne muscular dystrophy research are in green.

Site-specific endonucleases can modify the genome at a specific DNA locus, representing a promising tool to cure genetic disorders, including blood and muscular diseases [1]. Zinc finger nucleases (ZFNs) and transcription activator-like effector nucleases (TALENs) belong to the first generation of gene editing tools, comprising a customizable DNA-binding domain fused to a DNA cleavage module [2]. From the 1990s to the early 2010s, many scientists contributed to the identification of a microbial adaptive immune system against phage infection that relied on short repeated sequences (clustered regularly interspaced short palindromic repeats, CRISPR) and an RNA-guided DNA nuclease (CRISPR-associated protein 9, Cas9) [3,4,5,6,7] (Figure 1). The development of genome editing methods based on CRISPR/Cas9 technologies dramatically revolutionized the gene therapy field, rapidly expanding its therapeutic applications [8,9,10].

**Figure 2:**
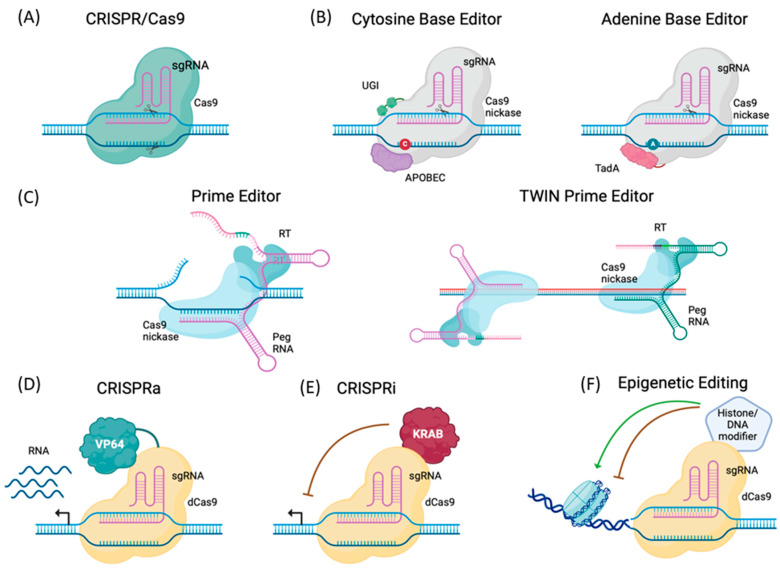
The expanding CRISPR/Cas9 toolbox. (**A**) CRISPR/Cas9, a gene-editing technology, is composed of two essential components: a guide RNA (sgRNA) to target a desired DNA locus, and the endonuclease Cas9 (CRISPR-associated protein 9). CRISPR/Cas9 leads to a double-stranded DNA break, allowing genomic modifications. (**B**) Base editing: nCas9 are fused to cytidine or adenosine deaminases (cytosine base editor or adenine base editor) to induce substitutions or base edits without inducing double-strand breaks. (**C**) Prime editing: nCas9 can be fused to a reverse transcriptase (RT) and extended prime editing guide RNA (pegRNA) (prime editor) to mediate targeted small insertions, deletions, and base-to-base conversions. Twin prime editing is an innovative iteration of prime editor protein based on two pegRNAs for programmable deletion, substitution, insertion, or inversion of larger DNA sequences. (**D**) dCas9 can be fused to transcriptional activators (CRISPRa) or (**E**) transcriptional repressors (CRISPRi) to precisely modulate gene expression. (**F**) dCas9 can be fused to epigenetic effector domains as histone/DNA modifier to alter the epigenetic state at precise DNA locations. *dCas9*—*dead Cas9*; *nCas9*—*nickase Cas9*.

The CRISPR/Cas9 system uses a chimeric single-guide RNA (sgRNA) to direct an endonuclease (Cas9) to specific DNA targets, where it generates double-strand breaks (DSBs) [9] (Figure 2A). These DSBs can be repaired by the cell through two competing pathways: the error-prone non-homologous end joining, which generates insertions and deletions at the cutting site, or the more precise homology-directed repair, which requires a DNA template to introduce specific modifications [11]. Pioneering CRISPR/Cas9 approaches have been successfully used in several clinical trials for lung cancer (NCT02793856) [12] and for β-hemoglobinopathies (NCT03655678, NCT03745287, NCT04443907), resulting in the first FDA-approved CRISPR therapy (www.fda.gov/news-events/press-announcements/fda-approves-first-gene-therapies-treat-patients-sickle-cell-disease, accessed on 8 December 2023). Despite these successes, it is becoming more evident that Cas9-induced DSBs can lead to genotoxic effects that include large insertions/deletions, chromosomal translocation, and chromothripsis [13,14,15,16], raising long-term safety concerns.

Base editing provides a more precise and efficient method for making single-base changes compared with traditional gene editing approaches [17] (Figure 2B). Potentially, around 60% of pathogenic point mutations could be corrected by base editors (Bes). Bes were developed by fusing a Cas9 nickase (nCas9) to a deaminase [18,19]. Unlike traditional CRISPR/Cas9, Bes do not generate DSBs; instead, the deaminase domain promotes base conversions at specific genomic locations upon sgRNA targeting. Cytosine Bes (CBE) can convert a C:G to a T:A base pair, while adenine Bes (ABEs) can convert A:T to G:C. Even if new Bes with less sequence constraints [20], C-to-G conversion capabilities (glycosylase base editors, GBEs [21,22]), and different editing windows have been developed [23], many pathological mutations cannot be targeted with this technology, including small and large deletions and multi-nucleotide changes. New editing tools have been generated to overcome these limitations, including prime editing [24], CRISPR/integrases [25], CRISPR/transposases [26], and large serine recombinases [27].

Prime editors (PE) can introduce specific mutations without inducing DSBs and perform insertions up to 44 bp [24] (Figure 2C). This system is composed of a nCas9 fused with a reverse transcriptase, and a prime editing gRNA (peg-RNA) that serves both as a guideRNA and a template to install the desired modification at a specific site. By using a pair of partially complementary pegRNAs, it is possible to increase insert size up to 110 bp or generate large genomic deletions (paired-peg, TWIN-PE) [28], though larger cassettes cannot be delivered using this system alone.

Serine site-specific recombinases like Bxb1 can integrate, invert, and excise large DNA fragments [29] by promoting the recombination of *attP* and *attB* sites. This reaction is irreversible and unidirectional, making this system ideal for stably integrating expression cassettes into the genome where one of the sites is present [30,31]. This observation prompted the development of novel editing tools like PASTE (programmable addition via site-specific targeting elements), which consists of a PE fused with Bxb1 [25]. PASTE can insert DNA payloads up to 35 Kb at specific genomic locations and could be beneficial for diseases involving large genes.

Apart from its capability to integrate, modify bases, or delete DNA sequences, the CRISPR/Cas9 system can be leveraged to modify gene expression (Figure 2D–F). Dead Cas9 (dCas9) can bind to specific DNA sequences by sgRNA targeting but has no nuclease activity. dCas9 can be combined with various enzymes like transcriptional repressors (CRISPR interference, CRISPRi) and activators (CRISPR activation, CRISPRa) to modulate gene expression over several orders of magnitude [32]. After binding, CRISPRi creates a steric hindrance that prevents transcription factors (TF) from attaching to the promoter region of the targeted gene, resulting in decreased expression. The system is more effective when transcriptional repressor domains (such as Krüppel-associated box, KRAB [33]) are fused to dCas9. Inversely, the CRISPRa system utilizes transcriptional activator domains (such as VP64, RTA, VP64, HSF1, and p65) to recruit RNA polymerase or TFs to promote and increase the expression of downstream genes [34,35]. Gene transcription can be altered at the epigenetic level by modulating DNA accessibility or chromatin structure at specific sites. Different epigenetic enzymes have been employed in combination with dCas9 to manipulate DNA methylation in gene promoters. For example, DNA methylases (DNMT) or demethylases (TETF1) have been used to modify the methylation status of specific CpG islands, therefore changing gene expression [36]. Chromatin accessibility can be altered by recruiting fusion Cas9 proteins containing histone modifiers such as histone acetylases (P300) that facilitate chromatin opening [37] or histone deacetylases (HDAC) that allow histones to wrap DNA more tightly [38].

In this review, we will summarize main preclinical and clinical applications of CRISPR-based technologies, focusing on monogenic blood disorders and muscular dystrophy, comparing advances in both fields, and highlighting current trends, difficulties, and challenges to overcome.

## 2. CRISPR-Based Gene Editing for Blood Disease

The only curative treatment for many inherited blood disorders is allogeneic transplantation of healthy hematopoietic stem/progenitor cells (HSPC). This procedure is limited by the availability of compatible donors and is associated with serious complications and life-threatening risks [39,40]. Gene therapy strategies based on autologous transplantation of ex vivo transduced HSPCs have been successfully developed. Despite the clinical benefits of these therapies, retro/lentiviral gene addition can result in subtherapeutic or unregulated transgene expression and carries an intrinsic risk of insertional mutagenesis. Hence, the ability to correct patient HSPCs using genome editing technologies represents a game changer for the field. HSPCs are routinely mobilized and isolated from patients and healthy donors. They can be modified ex vivo with different methodologies and reconstitute the entire blood system when transplanted [41]. These intrinsic properties of HSPCs, together with the extensive experience acquired through decades of lentiviral gene therapies, have accelerated the development of gene editing approaches for many hematological disorders. In this review, we will focus on two families of inherited blood diseases: β-hemoglobinopathies and primary immunodeficiencies.

### 2.1. β-Hemoglobinopathies

β-hemoglobinopathies are conditions affecting the β chain of hemoglobin, the main oxygen carrier in humans. In adults, the hemoglobin tetramer is composed of four chains—two α- and two β-globin subunits (HbA, α_2_β_2_)—both necessary to transport oxygen from the lungs to the tissues by the red blood cells (RBCs) [42]. β-hemoglobinopathies are the most frequent monogenic disease, affecting 400,000 births worldwide each year, the main forms being sickle cell disease (SCD) and β-thalassemia [43].

SCD is caused by an A-to-T mutation in the β-globin gene (*HBB*) resulting in the formation of hemoglobin S (HbS, β6^Glu→Val^) [44]. Under low-oxygen tension, HbS polymerizes, making erythrocytes more rigid and with a characteristic sickle shape. These sickled RBCs trigger hemolytic anemia and vaso-occlusion events, causing ischemic damage to tissues and leading to acute pain crisis and organ failure [45]. In contrast, β-thalassemia is caused by a wide range of mutations, including large deletions that decrease the amount of functional β-globin protein produced. The imbalance between β and α-chains causes precipitation of α-globin in RBCs, resulting in hemolysis and impaired erythropoiesis [46].

Despite different pathophysiology, both disorders can be ameliorated by the coinheritance of benign mutations that increase levels of fetal hemoglobin (HbF, α_2_γ_2_), as γ-globin can replace β-globin function and has antisickling properties [43,47]. For these reasons, many gene editing approaches for β-hemoglobinopathies rely on γ-globin reactivation. To achieve this goal, nucleases can target transcriptional repressors of γ-globin genes (*HBG1/2*) like B-cell lymphoma/leukemia 11A (BCL11a) or inhibitory regions in *HBG1/2* promoters. Many clinical trials were started in the past decade leveraging different CRISPR/Cas9-based tools (Table 1). Recently, the FDA approved the first CRISPR/Cas9 based treatment for severe SCD and transfusion dependent β-thalassemia (TDT)—Exa-cel (Casgevy) commercialized by Vertex Pharmaceuticals and CRISPR Therapeutics. This editing treatment consists of autologous HSPCs electroporated ex vivo with Cas9/gRNA mRNA to disrupt the erythroid-specific enhancer of BCL11a. Specifically, the BCL11A locus harbors three DNase I hypersensitivity sites (DHS), serving as erythroid-specific enhancers. These sites are situated at distances of +62, +58, and +55 kb from the transcriptional start site [48]. Among these, the DHS at +58 kb is crucial for BCL11a expression in erythroid cells [49,50]. Two clinical trials were initiated in 2018 for SCD (NCT05329649; NCT03745287; NCT05951205) and TDT (NCT05356195; NCT03655678) (Table 1). Initial findings reported successful engraftment of edited HSPCs (80% BCL11a alleles) one year post-treatment. This resulted in significant production of HbF, with a reduction in blood transfusions and vaso-occlusive events (in SCD patients) [51]. Two analogous strategies devised by Bioray (BRL-101) and Edigene (ET-01) to disrupt the +58 DHS of BCL11a are under clinical evaluation for TDT (BRL-101: NCT04211480; NCT05577312; NCT04205435; ET-01: NCT04390971; NCT05752123; NCT04925206) (Table 1). During the 18-month follow-up for BRL-101 (NCT04211480), two patients exhibited successful engraftment of modified HSPCs, achieving an editing rate of 85% in the bone marrow. This led to a clinically meaningful rise in HbF levels that translated to transfusion independence [52]. These results were also achieved in ten TDT patients [53]. Preliminary results from ET-01 also showed promising outcomes [54]. ZFNs were also utilized to disrupt the GATA-1 binding site in BCL11a locus in TDT (NCT03432364) (Table 1). Despite an initial success, a decline in γ-globin synthesis was observed in treated patients two years post-administration, requiring resumption of blood transfusions [55,56].

An alternative approach to reactivate HbF is to disrupt the BCL11a binding site on the γ-globin locus. In TDT patients, transplantation of HSPCs modified with CRISPR/Cas9 at the γ-globin promoter (ChiCTR2100052858; ChiCTR2100053406) increased HbF production, eliminating the necessity for blood transfusions [57,58] (Table 1). Alternatively, Cas12 nuclease has been successfully utilized to disrupt repressors binding sites on *HBG1/2* promoters [59]. This editing strategy showed high editing efficiency, with a 40% increase in HbF in vivo, and is currently under clinical investigation for TDT (NCT05444894, NCT06041620) and SCD (NCT04853576) (Table 1). Because of their superior safety profile and multiplexing properties, BEs have emerged as novel tools to reactivate γ-globin genes. This could be achieved by disrupting repressors binding sites at the *HBG1/2* promoters or by generating de novo DNA motifs for transcriptional activators. Antoniou et al. used BEs to generate several mutations associated with hereditary persistence of HbF and compared their impact on γ-globin expression [60]. In this study, the generation of a binding site for *KLF1* using ABE showed the greatest HbF reactivation in SCD cells. Beam Therapeutic also used ABE to generate a point mutation in *HBG1/2* promoters to restore γ-globin expression. This approach is currently being used in a clinical trial for SCD (NCT05456880) (Table 1).

Epigenetic activation of γ-globin has been proposed [35], both with zinc finger combined with transcriptional activator VP64 [61,62] and dCas9 fused with acetylase (p300) [37]; however, their γ-globin reactivation efficiency is lower compared with genome editing approaches.

Direct correction of the SCD mutation in *HBB* has been attempted, leveraging the homology directed repair pathway. Different corrective templates have been used as DNA donors in combination with CRISPR/Cas9, including adeno associated virus (AAV) [63] or single-stranded oligodeoxynucleotides (ssODN) [64]. This approach was further developed [65] to initiate a clinical trial for SCD (NCT04819841) [66] (Table 1). However, Graphite Bio halted the trial in early 2023 after observing complications in the first patient dosed, including prolonged low blood cell counts requiring transfusion and growth factor support.

Based on similar methods, gene replacement approaches that rely on the integration of the β-globin gene into the α-globin locus have been devised [67,68]. These studies successfully restored HbA expression in thalassemic and SCD erythroblasts. Such approaches might benefit patients with large deletions spanning the γ- and β-globin locus where HbF reactivation is not a viable therapeutic option.

BEs cannot directly correct the A>T mutation present in SCD. However, ABEs have been used to modify the SCD allele to a non-pathogenic β-globin variant (β6^Glu→Ala^, Hb-Makassar) [69,70]. An engineered ABE nuclease edited between 45% to 80% of the SCD alleles in patient HSPCs, resulting in up to 72% of Hb-Makassar production in differentiated erythroid cells [70]. The diverse mutational spectrum in β-thalassemia poses challenges to the development of corrective therapies. Hardouin et al. proposed a base editing approach to treat a common disease-causing point mutation in *HBB* (IVS1-110 [G>A]) that efficiently rescued β-globin production [71]. Because of their precise editing activity, PEs can correct the SCD mutation [24]. Everette et al. delivered an optimized ePegRNA and PE3max as mRNA into SCD-HSPCs and achieved up to 41% of allele correction. Analysis of engrafted cells isolated 4 months after transplantation showed expression of HbA in 42% of human red cells [72]. Rather than relying on the ex vivo correction of HSPCs, Li et al. developed a novel in vivo modification strategy to correct the underlying SCD mutation. After HSCP mobilization, a helper-dependent adenoviral vector (HDAd) encoding the PE system was administrated intravenously in an SCD mouse model (CD46/Townes). Viral transduction corrected up to 40% of SCD alleles in vivo and increased HbA synthesis to similar levels [73]. While safety aspects of this delivery method need to be further explored, the direct modification of HSPCs in vivo eliminates the requirement of myeloablation/transplantation, a critical limitation for many gene editing therapies.

Overall, CRISPR/Cas9-derived tools have generated innovative and efficient treatments for β-hemoglobinopathies. These studies are paving the way for a broader application of this ground-breaking technology to many other genetic disorders.

### 2.2. Primary Immunodeficiencies

Primary immunodeficiencies are rare diseases comprising more than 130 genetic disorders that affect the function and/or development of the immune system [74]. Some of these diseases, such as adenosine deaminase-severe combined immunodeficiency (ADA-SCID) and X linked-SCID (SCID-X1), have been successfully treated with viral gene therapy [75,76]. However, retroviral and lentiviral vectors carry the intrinsic risk of oncogenic insertional mutagenesis, and therefore alternative strategies are needed. Moreover, many genes involved in primary immunodeficiencies require a thoroughly regulated expression and would benefit from a gene editing approach. Because pathological mutations are rare and scattered along the gene body, most editing strategies for these disorders rely on the targeted integration of a corrective cDNA at the endogenous locus. For many immunodeficiencies, corrected cells have a selective advantage over mutated ones. Even if gene knock-in is not efficient in HSPCs, low correction levels could be sufficient to treat the disorder in patients. In this section, we will describe genome editing approaches developed for a subset of primary immunodeficiencies.

X-linked hyper-IgM syndrome (XHIM) is caused by mutations in the CD40 ligand (CD40L) gene that result in ineffective antibody production, recurrent infections, and autoimmunity in male infants. Ectopic expression of CD40L has been associated with lymphoma in mice [77], therefore lentiviral approaches would not be recommended for this disease. Proof-of-principle studies were performed using Cas9 and other nucleases to integrate CD40L cDNA under the control of its endogenous promoter in primary T cells [78,79] and in healthy HSPCs [80]. Importantly, CD40L activity was restored in gene-edited cells in vitro and in vivo, without signs of aberrant differentiation. Recently, large-scale good manufacturing practice (GMP)-compliant production processes have been developed, bringing this therapy closer to clinical application [81].

*RAG1* is another gene involved in primary immunodeficiencies that requires tight regulation. Recombination activating genes (RAGs) are involved in V(D)J recombination and are required for correct lymphocyte development and function. A clinical trial using a lentiviral approach is currently underway (NCT04797260); however, constitutive and/or heterogenous expression of RAG1 raises safety concerns about potential immune dysregulation [82]. Using Cas9 and an AAV6 with a corrective cDNA, Castiello et al. restored RAG1 expression and activity in patient HSPCs, leading to the generation of mature T and B cells in transplanted mice [83].

Chronic granulomatous disease (CGD) is a group of genetic disorders in which neutrophils are unable to kill certain bacteria and fungi. The X-linked form of the disease (X-CGD) is caused by mutations in the *CYBB* gene. Different editing strategies were proposed to correct X-CGD in HSPCs, using nucleases with AAV6 vectors or ssODN as donor templates [84,85,86]. These studies indicated that preserving the first intron of *CYBB* is crucial to achieve a normal level of expression and restore effective ROS production [86]. Unfortunately, gene correction rates need to be extensively optimized to achieve therapeutic effects as corrected X-CGD cells do not have a selective advantage in vivo [87].

Most p47-CGD patients carry a specific deletion (ΔGT) in exon 2 of the *NCF1* gene, making this form of CGD a good candidate for gene editing therapies. Two studies have shown correction of the ΔGT mutation using nucleases in combination with a DNA template [88,89], however efficiency must improve to reach therapeutic benefits in preclinical models.

Severe congenital neutropenia (SCN) is a rare inherited disease that impairs neutrophil maturation and is frequently caused by autosomal-dominant mutations in the *ELANE* gene. Production of an abnormal protein results in neutropenia, despite the presence of one intact *ELANE* allele. Induction of frameshift alleles with CRISPR/Cas9 can trigger non-sense mediated decay and potentially relieve neutrophil maturation arrest [90,91]. An allele-specific knockout strategy to precisely target the mutated copy of *ELANE* has been proposed. By targeting heterozygous sites of single-nucleotide polymorphisms (SNPs) associated with *ELANE* mutations, Sabo et al. were able to rescue neutrophil development and function [92].

CD3δ-SCID is caused by biallelic mutations in the autosomal *CD3D* gene that result in a profound deficiency of circulating mature T cells, often leading to infant mortality. CD3D is essential for the productive assembly of TCR complexes, playing an important role in T cell maturation [93]. In a recent study, McAuley et al. delivered ABE to CD3δ-SCID HSPCs to correct the disease-causing *CD3D* c.202C>T mutation [94]. High editing efficiency was achieved in these cells, together with functional correction and restoration of T cell maturation. Edited cells persisted 16 weeks in xenografts and displayed normal T cell receptor repertoires, making this approach a promising treatment for CD3δ-SCID.

Wiskott–Aldrich syndrome (WAS) is an X-linked disorder caused by mutations in the *WAS* gene that accounts for approximately 3% of all primary immunodeficiencies [95]. Clinical manifestations depend on the underlying mutation, ranging from mild to severe persistent thrombocytopenia, autoimmunity, increased susceptibility to infections, or congenital neutropenia [96]. Lentiviral gene therapy has been successfully used for WAS [97,98], but bleeding and autoimmune episodes were only partially resolved by the treatment. Several knock-in strategies have been developed using different nucleases and DNA donors in pluripotent stem cells and cell lines [99,100]. Recently, Rai and al. restored WAS function in patient HSPCs by targeting WAS cDNA to its endogenous locus, using Cas9/gRNA and AAV6 [101]. A side-to-side comparison was also performed between genome edited and lentivirus transduced cells. Interestingly, transduced cells showed improved functionality but to a much lesser extent compared with gene edited cells. Limited correction of B-cells, platelets, and myeloid cells was observed in the lentiviral group, possibly due to suboptimal expression of WAS protein. In light of these promising results, preclinical studies are on the way to further assess the safety and efficacy of this therapeutic approach [102].

## 3. CRISPR-Based Gene Editing for Duchenne Muscular Dystrophy

Similarly to blood diseases, the last decade witnessed the advent of CRISPR-base gene editing in neuromuscular disorders, notably in the Duchenne muscular dystrophy (DMD) field. From the rapid generation of helpful cellular and animal models to pre- and clinical development of innovative therapeutic options, CRISPR gene editing technology has revolutionized DMD research.

**Figure 3:**
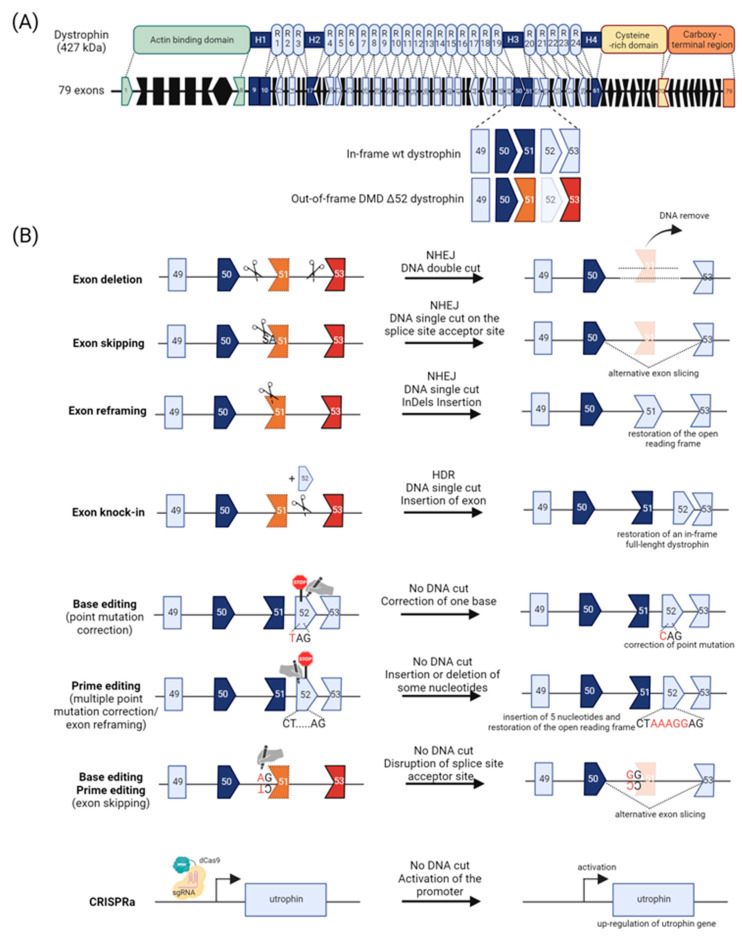
Dystrophin structure and CRISPR/Cas9 strategies for DMD. (**A**) Structural organization of the full-length wild-type dystrophin composed of 79 exons: the actin-binding N-terminal domain, the hinge domains (H1–H4), a rod domain consisting of 24 spectrin domains (R1-R24), and a cysteine-rich domain (CRD) next to a carboxy-terminal domain (CTD). The out-of-frame DMD Δ52 mutation (deletion of exon 52) is one of the most common mutations in DMD patients. (**B**) CRISPR/Cas9 mediated therapeutic strategies for DMD: NHEJ method (exon deletion, exon skipping, exon reframing), HDR method (exon knock-in), base editing, prime editing, and CRISPR activator.

DMD is a fatal X-linked neuromuscular disorder characterized by severe and progressive muscle weakness and wasting due to degeneration of skeletal, smooth, and cardiac muscle [103]. Affecting more than 300.000 males worldwide, with a 1:5000 incidence in male newborns [104], DMD is the most prevalent genetic muscular disorder in humans. This orphan disease is caused by a variety of different mutations (mainly deletions (≈68%), point mutations (≈20%), and duplications (≈11%)) in the *DMD* gene (OMIM 300377, Xp21.2-p21.1) [105], known as the largest human gene (79 exons over 2.2 Mb of genomic DNA) (Figure 3A). The *DMD* gene encodes for dystrophin (Uniprot P11532) [106], an essential 427 kDa cytoplasmic protein critical for the maintenance of the biomechanical properties of fiber strength, flexibility, and stability in skeletal muscle [107]. Acting as a molecular shock absorber, the dystrophin protein establishes a mechanical link between the extracellular matrix and the actin cytoskeleton through the dystrophin-associated protein complex (DAPC) and allows myofibers to cope with repeated cycles of muscle contraction and relaxation. The absence of dystrophin destabilizes the DAPC and consequently weakens the link between cytoskeleton and extracellular matrix, triggering muscle cell dysfunction, muscle cell necrosis, and subsequent dystrophic tissue changes resulting in fibro-fatty replacement of skeletal muscle. Affected boys typically develop the first signs of motor dysfunction around the age of 2–3 years with abnormal gait, weakness in proximal muscles and calf muscles, pseudo hypertrophy, and difficulties in running, jumping, and climbing stairs. These symptoms progress relentlessly, loss of ambulation occurs around the age of 10–12 years [108], followed by progressive generalized muscle paralysis including respiratory insufficiency and cardiomyopathy [109]. Most patients decease in young adulthood, with a median survival of 28.1 years (95% CI 25.1, 30.3) [110], as a result of cardiac and respiratory failure. Despite exhaustive clinical attention for respiratory support, management of cardiac complications, and corticosteroid treatment, there is currently no cure for this devastating disease. The urgency to seek a cure for DMD has resulted in parallel efforts to develop exon skipping [111,112], termination codon read through [113] and dystrophin gene therapy [114], and non-dystrophin strategies [115]. Among all, anti-sense oligonucleotide (ASOs)-based exon skipping therapies aiming to restore a functional reading frame by skipping one or more exons to produce a functional dystrophin, are one of the most promising therapeutic options for DMD. Several exon-skipping drugs were recently approved and, despite their success, new generation of ASOs with improved chemistries, distribution, and efficiency such as the peptide-conjugated phosphorodiamidate morpholino oligomer (P-PMO) [116] and Tricyclo-DNA (tcDNA) [117] are currently required and under investigation in clinical trials for DMD. Acting at the DNA level, CRISPR/Cas9 technology offers novel and promising opportunities in DMD gene therapy.

### 3.1. CRISPR/Cas9 and Generation of DMD Study Models

To study the pathophysiology of the disease and to evaluate the efficacy of therapies prior to clinical trials, cellular and animal models are mandatory. Only few immortalized patient-derived muscle cell lines are currently available [118] as DMD muscle biopsies are difficult to obtain. CRISPR/Cas9 technology allowed researchers to directly generate human DMD lines with specific genetic mutations (Table 2). In the past years, several novel immortalized DMD muscle cell lines were generated with the deletion of exons 50 [119], 51 [119], 52 [119,120], and 53 [119]. Recently, Tremblay’s lab produced DMD lines with specific point mutations in exons 9, 20, 35, 43, 55, and 61 using prime editing [121]. These cell lines and associated subsequent assays [120,122,123] represent powerful tools to evaluate and optimize therapeutic strategies, screen for effective drugs, and gain insights in the disease pathophysiology.

Similarly, CRISPR/Cas9 offered a simple and efficient method to generate new animal models of DMD (Table 2). The *mdx* mice is the most widely used DMD model and presents a spontaneous point mutation in exon 23 of the *Dmd* gene, resulting in a premature stop codon [124]. However, the *mdx* mouse develops a moderate and nonprogressive myopathy, with a slightly shorter lifespan, and fails to recapitulate many symptoms observed in DMD patients. Several murine models carrying specific exonic deletions were generated using CRISPR/Cas9, such as ΔEx52 [125], ΔEx50 [126], ΔEx45 [125], ΔEx44 [127], ΔEx43 [125], or the large deletion ΔEx8-34 [128]. Murine DMD models suffering from a non-sense mutation in the *Dmd* exon 20 [129] and frameshift mutation [130] were also produced by CRISPR and base editing. Importantly, all these strains phenocopied the *mdx* mice and suffered from the same limitations. Moreover, these DMD models are of limited use to test human sequence-dependent therapies such as exon skipping and gene editing approaches. Consequently, humanized DMD mouse strains (hDMD mouse) such as the hDMDΔ52/*mdx* [131] and the hDMDΔ45/*mdx* [132] were produced. Partially humanized mice were similarly generated, replacing exon 45 and its adjacent introns with the human sequences ((hEx45KI-mdx44) [133]. Although splicing mechanisms might be different, these CRISPR-generated DMD models provide unvaluable in vivo platforms to assess human sequence-dependent exon 44, 45, and 51 skipping drugs and gene editing approaches.

Rat models of DMD recapitulate more faithfully the human DMD pathophysiology, allow behavioral experiments with high statistical power, and may represent a useful alternative to mice. The first Dmd*^mdx^* rat was generated by TALEN [134] and suffered from a severe muscle and cardiac pathology, reflecting some lesions and functional abnormalities observed in DMD patients. Using a two sgRNA pairs approach, the Relaix’s lab successfully generated the R-DMDdel52 rat model, with a premature stop codon in exon 52 of the rat Dmd gene [135]. Nakamura et al. also reported the generation of a Dmd ΔEx3-16 rat using SpCas9 and two sgRNA [136], and a Dmd ΔEx45 rat model was recently produced [137]. In the near future, humanized DMD rat models may certainly serve preclinical drug development.

With an intermediate size and low maintenance costs, rabbit is another interesting animal model of DMD. The first DMD rabbit was produced by co-injecting SpCas9 mRNA and two gRNAs targeting the DMD exon 51 into rabbit zygotes [138]. This DMD ΔEx51 rabbit model suffered from reduced dystrophin expression, histological defects, impaired physical activity, cardiomyopathy, and premature death. At the crossroads between small and large models, this novel rabbit DMD model could be valuable for preclinical studies.

In addition to rodent models, larger animal models of DMD such as pig, dog, and monkey have also been produced using CRISPR systems. Porcine models have a number of practical advantages and are phenotypically and phylogenetically closer to humans. Several porcine models of DMD, such as the DMD ΔEx52 pig [139], the DMD ΔEx27 Chinese Diannan pig [140], or the DMD mEx13 [141], have been described and exhibited a severe muscular dystrophy similar to human DMD but suffered from a faster disease progression. DMD pigs are an attractive in vivo animal model, but their long gestational period, costly maintenance, and reduced survival makes their preclinical use challenging.

The golden retriever muscular dystrophy (GRMD) dog [142] and several other breeds [143,144] suffered from spontaneous DMD genetic defects and closely mimicked the human disease [145]. Oh et al. reported the generation of a DMD dog with a 57 bp deletion in exon 6 by nuclear transfer using CRISPR/Cas9-edited somatic cells [146]. While expensive and limited by the low number of phenotypically variable individuals, DMD dogs represent a useful large animal model for preclinical studies.

Monkeys have mostly been used for safety evaluation and pharmacological studies in the DMD field. Recently, the first DMD rhesus monkeys (*Macaca mulatta*) model with various InDel mutations in exon 4 and 46 was generated [147]. These animals showed dystrophin reduction and some histological defects. To date, this study is the only published preliminary assessment of a DMD monkey model.

The ideal animal model should display a robust phenotype over generations, have the same genetic defect, and mimic key hallmarks and progression of the human pathology. None of the 60 animal models of DMD previously described fulfilled these requirements. Each animal model presents key advantages and limitations and should be used for specific applications. As an example, murine models are indicated for in vivo drug screening, while dogs and monkeys are appropriate for assessing immune responses and toxicology. CRISPR technologies have facilitated the generation of novel animal models of DMD, speeding up preclinical development of therapeutic solutions and their translation to DMD patients.

**Table 2:** CRISPR-mediated animal models of DMD.

	Model	DMD Mutation	Target	Nuclease	Advantages	Limitations	Reference
**Cell**	human immortalized myoblasts cell line	ΔEx50	I49 and I50	SpCas9	- Low cost - Easily maintainable - Wide number of mutations - Human genetic context - High high-througput screening	- Cell type number limited - Does not recapitulate the human disease	[119]
		ΔEx51	I50 and I51				[119]
		ΔEx52	I51 and I52				[119,120]
		ΔEx53	I52 and I53				[119]
**Animal**	Mouse	Ex20	Ex20	BE3	- Low cost - Easily maintainable - Wide number of mutations - High statistical power - Extensive data/littérature - Accepted protocols (TREAT-NMD).	- Only mildly affected - Does not recapitulate the human disease	[129]
		Ex23	Ex23	CjCas9			[130]
		ΔEx43	I42 and I43	SpCas9			[125]
		ΔEx44	I43 and I44				[127]
		ΔEx45	I44 and I45				[125]
		ΔEx50	I49 and I50				[126]
		ΔEx8-34	I7 and I34				[128]
	Humanized Mouse	ΔEx45	I44 and I45				[132,133]
		ΔEx52	I51 and I52				[131]
	Rat	ΔEx52	I51 and I52		- Eeasily maintainable - Severe skeletal/cardiac muscle phenotype - Allow behavioural experiments - High statistical power	- Higher cost than mice (5×)	[135]
		ΔEx3-16	Ex3 and Ex6	SpCas9			[136]
	Rabbit	Ex51	Ex51	SpCas9	- Severe muscle dystrophy - Severe cardiomyopathy - Intermadiate body size - Relatively short gestational period	- Expensive to maintain - Lack of accepted protocols	[138]
	Pig	Ex13	Ex13	BE3, hA3A-BE3	- Severe muscular dystrophy - Anatomy, size and physiology closer to humans	- Expensive to maintain - Long gestational period - Accelerated dystrophy compared to human - Stress may induced sudden death - Lack of accepted protocols	[141]
		Ex27	Ex27				[140]
		Ex52	Ex52				[139]
	Dog	Ex6	Ex6	SpCas9	- Severe muscular dystrophy - Severe cardiomyopathy - Anatomy, size and physiology closer to humans	- Expensive to maintain - High variability - Low statistical power	[146]
	Monkey	Ex4 or Ex46	Ex4 or Ex46	SpCas9	- Greatest similarity to humans	- Expensive to maintain - Long gestational period - Recent model, lack of accepted protocols	[147]

### 3.2. CRISPR-Based Therapies: From Preclinical to Clinical Trials for DMD

The simplicity, versatility, and precision of CRISPR-/Cas9 offer novel and promising therapeutic opportunities for DMD. Acting at the DNA level, CRISPR/Cas9 has the potential to permanently repair the genetic cause of DMD. Here, we report the most advanced CRISPR/Cas9 therapeutic interventions to correct and restore dystrophin expression, with a focus on preclinical in vivo studies (Figure 3B).

### 3.3. NHEJ-Mediated Exon Deletion

More than 60% of DMD patients suffer from exon deletion and 11% of all DMD cases are due to exon duplications [104,148]. Depending on the genetic defect, deletion of one or more exons restores the reading frame, resulting in a shorter but functional dystrophin protein. Many in vitro [149,150] and in vivo studies have demonstrated that the CRISPR/Cas9 system and appropriate pairs of sgRNA can be used to induce exon(s)/intron(s) excision and restore the DMD reading frame (Figure 3B). In 2016, three independent studies demonstrated in neonate and adult *mdx* mice that SpCas9 or SaCas9 and dual sgRNA treatment delivered by rAAV8, 9 could efficiently delete the mutated exon 23, restore dystrophin expression and consequently improve muscle morphology, and enhance skeletal and cardiac muscle function [151,152,153]. The seminal studies provided evidence for permanent and efficient gene correction in dystrophic animals. Importantly, such an approach can be sustained for up to 19 months in murine animals without inducing serious adverse effects [154,155,156]. Correction of other DMD genetic defects, including deletions ΔEx45 [132], ΔEx52 [157], and ΔEx52-53 [158,159] or the duplication of Ex18-30 [160], demonstrated the potential of this CRISPR-based approach to eliminate mutated exon, point mutation, and exonic duplication in different animal models of DMD, notably in large animal such as pigs [161] and dogs [162], paving the way for clinical translation. Deletions and duplications cluster in two main hotspots in the DMD gene, located at exons 45–55 and 3–9. Following the initial work from Ousterout et al. and the successful transplantation of CRISPR/Cas9-corrected Δ45-55 patient cells into immunodeficient mice [163], Young et al. generated a large 45–55 exonic deletion in an hDMD ΔEx45/*mdx* mice model [132]. Treatment resulted in the expression of a truncated Δ45-55 dystrophin in muscle animal, demonstrating the clinical relance of the CRISPR/Cas9 platform. Similarly, Duchêne et al. demonstrated the restoration of the DMD ΔEx47-58 reading frame and in vivo dystrophin expression in hDMDΔEx52/*mdx* mice after AAV9 administration delivering SaCas9 and 2 sgRNA targeting the exon 47 and 58 [157]. Whereas such innovative gene editing strategy is applicable to ~60% of DMD patients, it should be noted that efficiency is correlated with deletion size and that multi-exon deletions generate a shorter and potentially less functional dystrophin.

### 3.4. NHEJ-Mediated Exon Skipping

More than 70% of the DMD population is amendable to exon-skipping strategies aiming to excise one specific exon to restore a functional open reading frame and produce a truncated but functional dystrophin protein [164]. Exon-skipping approaches are mutation-dependent and specific for subsets of the DMD patient population [165]. Disruption of splicing donor or acceptor sites with a single gRNA and CRISPR/Cas9 introduces InDels, abolishes splice element function, and leads to the skip of the targeted exon (Figure 3B). Several in vitro studies in human DMD myoblasts and human iPSCs reported efficient exon 43, 51, and 53 skipping using this approach [166]. In animal models of DMD, Olson’s lab induced skipping of exon 51 in neonate ΔEx50/*mdx* mice using a dual AAV9 approach to deliver SpCas9 and three copies of the same gRNA targeting a region adjacent to the exon 51 splice acceptor site [126]. Systemic treatment conducted a widespread dystrophin expression in skeletal, respiratory, and cardiac muscles, resulting in histological correction of the dystrophic defects and significant increased muscle strength. Similar results were obtained in a humanized ΔEx50/*mdx* model [167]. The same group reported similar results in ΔEx50-MD dogs, with a restoration of dystrophin levels ranging from 3 to 90% of normal, reaching 92% in the heart [162]. Correction of other exon deletions such as ΔEx43, ΔEx44, ΔEx45, and ΔEx53 were also studied in animal models [125,127,168,169]. Of interest, these studies demonstrated that the amount of sgRNA delivered to the muscle was critical for efficient editing in vivo [127] and provided evidence for the long-term benefits of single-cut gene editing therapy, even under conditions of stress [168]. Compared with CRISPR-mediated exon deletion using two sgRNA, single-cut exon skipping is a safer approach as it reduces potential off-target effects and DNA damage. Importantly, such exon-skipping strategies may also result in exon reframing.

### 3.5. NHEJ-Mediated Exon Reframing

Similarly to single-cut exon skipping, the exon reframing approach relies on the use of a single gRNA targeting a frameshift mutation and the subsequent generation of small InDels by NEHJ to restore a functional reading frame. In theory, small indels generated upstream of a deleterious mutation have a 1 in 3 probability of restoring the reading frame. If successful, this approach will restore a larger dystrophin protein compared with exon skipping (Figure 3B). Several in vitro studies [163,170] demonstrated the efficacy of this CRISPR strategy. Positive results were observed in vivo in ΔEx50/*mdx* mice after AAV9 delivery of CRISPR components to restore the open reading frame of exon 51. Muscle function improved toward wildtype levels even with low gene editing efficiency (8%-12%), resulting in 27–45% of dystrophin expression in skeletal, respiratory, and cardiac muscles [171]. This approach was applied to several *mdx* mice, including mEx23 [130], ΔEx50 [126], hΔEx43 [125], hΔEx44 [127], hΔEx45 [125], and hΔEx52 [125] *mdx* mice. Interestingly, Min et al. compared both exon skipping and reframing strategies for three DMD mutations and concluded that exon reframing was more efficient than exon skipping in restoring dystrophin [125].

### 3.6. HDR-Mediated Gene Correction

Unlike NHEJ-based strategies, HDR-mediated gene editing has the potential to restore the full-length dystrophin and to correct mutations in the N- and C-terminal region of the protein (Figure 3B). The first in vivo proof-of-concept of this approach was provided in 2014 [172] by zygotic injection of SpCas9/sgRNA and an exogenous DNA template in *mdx* mouse embryos. This seminal work demonstrated that ~17% of gene editing was sufficient to restore dystrophin expression to wild-type levels. Consequently, several investigations have attempted to correct mutations in exons 23 [173,174], 44 [170], and 53 [158] of the Dmd gene using similar knock-in approaches. In *mdx^4cv^*, intramuscular injection of an AAV6 delivering Cas9/sgRNA and a donor template rescued dystrophin production to 1.8–8.4% of the wild-type level following a 0.18% gene editing efficiency [158]. In a GRMD dog model, intramuscular injection of Cas9 components and a corrective ssODN restored dystrophin expression to 6–16% of normal levels [175]. The NHEJ-based knock-in homology-independent targeted integration (HITI) approach has also been leveraged to correct genetic defects [176,177]. By including two Cas9 cleavage sites flanking the donor sequence, HITI allows efficient integration of a DNA template at the targeted genomic site. Pickar-Oliver et al. successfully knocked-in exon 52 and super-exon 52–79 in an hDMDΔ52 mouse model [131]. Despite the low efficiency, these results demonstrated that HITI can restore the dystrophin protein in vivo. While the accuracy of HRD- and HITI-based strategies is appealing, the major limitation of these approaches is their low efficiency. Additionally, inverted/unwanted integration of the DNA donor may occur, resulting in potential alteration of dystrophin expression. Extensive optimization and safety studies will be required to advance these therapies to the clinic.

### 3.7. Base Editing

Beyond traditional NHEJ- and HDR-based CRISPR/Cas9 systems, generation of catalytically impaired Cas9 (D10A) fused to a base-modifying enzyme introduced the first iteration of a more precise and safer gene editor [23] (Figure 2B). Cytosine base editors (CBEs) [19] and adenine base editors (ABEs) [18] respectively allow C:G to T:A and A:T to G:C base pair transitions without DSBs, DNA donor template, or HDR, offering a promising editing avenue to precisely correct single point and non-sense mutations (Figure 1 and Figure 2), accounting for ~27% of the DMD cases [178]. Described as safer than CBEs due to a higher specificity and lower off-target activity [179,180], several ABE systems were successfully used in different mouse models of DMD (Figure 3B). Ryu et al. used trans-splicing to deliver a full ABE with two AAV9s to correct a point mutation generating a premature stop codon in exon 20 of a murine Dmd gene [181]. Eight weeks post infusion, dystrophin expression was detected in 17% of the skeletal myofibers. Similarly, Hu and colleagues developed a single AAV9-iABE-NGA systemic treatment designed to correct a non-sense mutation in exon 53 of the *mdx*^4cv^ mouse [182]. A widespread restoration of dystrophin expression and subsequent histopathological and functional benefits were noted 10 weeks post-injection. Importantly, therapeutic benefits were maintained 10 months after injection, with a near complete rescue of dystrophin in the heart and 15% rescue in skeletal muscle fibers. By altering splice sites, BEs can promote exon skipping and correct mutations and deletions in the *DMD* gene. Systemic administration of AAV9s delivering CBE and sgRNA designed to skip exon 4 in Dmd^E4^ mice resulted in efficient dystrophin restoration and rescue of muscular dystrophy phenotype [183]. Similar results were obtained by Chemello and al. following intramuscular injection of dual AAV9s encoding ABE components as a split-intein trans-splicing system promoting exon-50 skipping in ∆Ex51 mice [184].

While these preclinical studies demonstrate feasibility and efficacy of BEs for many DMD mutations, delivery remains a major challenge. Due to their large size, BEs require dual-vector delivery and increased viral doses associated with immunological and toxicological concerns. A novel optimized all-in one BE solution able to fit into a single AAV [185] may lead to further the preclinical development of these innovative therapies for DMD.

### 3.8. Prime Editing

The versatility of prime editing has opened up an attractive horizon for the treatment of various genetic defects such as DMD [186] (Figure 2C and Figure 3B). Olson’s lab applied first prime editing to ∆Ex51 DMD patient-derived iPSCs to reframe exon 52 and successfully restored dystrophin expression to 24.8–39.7% of healthy levels [184]. Similarly, Tremblay’s lab used PE to induce substitutions, insertions, and deletions in the splice donors for exons 51 and 53 to correct frameshift mutations in the *DMD* gene carrying exon 52 or exons 45 to 52 deletions [187] and to correct point mutation in the *DMD* gene [121,188]. Recently, Anzalone et al. demonstrated that twin PE systems can precisely excise exon 51 in the dystrophin gene in vitro [28], paving the way for an innovative gene editing solution for DMD.

While this technology has the potential to correct a large variety of DMD mutations, further in vivo applications are required to progress and translate this approach to patients and, similarly to base editing, size constraints remain a challenge to be solved for in vivo delivery.

### 3.9. CRISPR/Cas9 Activation

In addition to enabling the engineering of eukaryotic genomes, recent alterations to the CRISPR/Cas9 system have provided opportunities for regulating gene expression [189] (Figure 2D). Modulation of disease-modifier genes can provide therapeutic solutions applicable to all DMD patients irrespective of their dystrophin mutation [115,190]. Utrophin (*UTRN*), an autosomal and functional paralogue of dystrophin expressed at the sarcolemma of regenerated myofibers [191,192], has the potential to compensate for the primary defect in DMD [193]. Seminal preclinical studies demonstrate that expression of UTRN suppresses functional signs of dystrophinopathy in a dose dependent manner [194] without toxicity [195], and strongly support UTRN as a functional surrogate for dystrophin [190,196]. Using *CRISPRa* (dCas9-VP160) targeted to the *UTRN* promoter, Wojtal et al. successfully modulated UTRN expression in DMD myoblasts [197]. Liao and colleague demonstrated a three- four-fold in vivo increase in UTRN levels in *mdx* mice using a different CRISPRa system [198] (Figure 3B). While these preclinical studies emphasize the potential of CRISPR-based UTRN upregulation for DMD, such approaches require long-term efficacy and toxicology in vivo assessment.

Recently, Cure Rare Disease, Inc. launched the first CRISPR-based clinical trial for DMD (NCT05514249) [199]. The n-of-1 clinical trial involved a single 27-year-old DMD patient suffering from an exon 1 deletion, lacking the dystrophin muscle isoform (Dp427m). Phase 1 aimed to assess the safety and preliminary efficacy of CRD-TMH-001, an AAV9-dCas9-VP64 delivered intravenously at a 1×10^14^ vg/kg dose and designed to upregulate cortical dystrophin through the conserved Dp427p promoter. Six days after receiving the therapy, the patient showed signs of mild cardiac dysfunction and pericardial effusion, leading to cardiac arrest two days later. Post-mortem examination revealed severe acute-respiratory distress syndrome (ARDS) with diffuse alveolar damage. Investigators concluded that the high dose of AAV triggered the adverse event and not the CRISPR therapy itself. Despite remarkable success in gene therapy, lethal immunotoxicity in high-dose systemic AAV therapy was previously reported in treated DMD, SMA, and XLMTM patients [200]. This first reported case of severe ARDS linked to an AAV product provided a painful but valuable lesson that will help de-risk the translation of gene therapies for future patients and remind us of the challenges we still need to overcome.

## 4. From the Same Technology to Different Stages: A Comparison of CRISPR-Based Strategies for Blood and Muscle Fields

While CRISPR/Cas9 strategies for blood disorders rapidly advanced from early in vitro studies to clinical trials, gene editing for neuromuscular diseases has not yet reached similar milestones. Clinical translation of the same technology may progress differently across medical fields because of disease-specific limitations.

While hematopoietic stem cells can be purified, corrected ex vivo, and re-infused into the patient [201], CRISPR/Cas9 components need to be systemically delivered in order to reach the muscles. For the treatment to be effective, AAV vectors carrying the editing machinery must transduce skeletal, respiratory, and cardiac muscle tissues specifically and efficiently. This requires the choice of adequate serotypes and appropriate dosing, balancing efficacy considerations and risks associated with systemic AAV vector delivery [202]. From the administration route to vector manufacturing, neuromuscular diseases currently face a higher level of complexity compared with blood disorders, explaining why CRISPR/Cas9 therapies have not yet reached the same clinical stages. While extensive optimization efforts are currently ongoing to address such challenges [202], it should be noted that DMD will greatly benefit from the clinical success of CRISPR in blood disorders and future long-term follow-up studies.

A major bottleneck to clinical translation for novel DMD therapies is the lack of suitable animal models. The widely used *mdx* mouse [124] does not recapitulate the human disease and does not offer the appropriate genetic context for sequence-based approaches, such as exon skipping, strop codon read through, and CRISPR/Cas9 strategies. CRISPR has been an incredible tool to produce novel humanized animal models for DMD, speeding up the translation of sequence-based approaches to patients.

## 5. Conclusions: Challenges and Current Trends

In recent years, CRISPR has emerged as a revolutionary technology with the potential to permanently and precisely edit any DNA locus and correct genetic errors. From CRISPR-Cas to base and prime editors, the CRISPR tool box has generated innovative preclinical models and corrective strategies for many genetic disorders, bringing more breakthrough therapies closer to the patients. The last decade has witnessed the rapid development of this technology for monogenic blood disorders, from the early proof-of principle studies to the first regulatory approval of a CRISPR-based therapy for SCD and TDT. To date, there are currently 94 clinical trials for hereditary and non-hereditary diseases based on CRISPR technologies (https://clinicaltrials.gov). Nevertheless, as illustrated by blood and neuromuscular disorders, there are still many hurdles to overcome to expand clinical applications of gene editing therapies, including long-term safety and off-target activity and also cost, accessibility, and manufacturing. Some concerns are specific to the targeted organ system.

Despite the success of Casgevy in SCD and TDT and many promising results from undergoing clinical trials, we still do not know the long-term efficacy and safety of these treatments. While effective at reducing vaso-occlusive events and transfusion dependency in SCD, we need long-term follow-up studies to establish whether Casgevy can also decrease disease burden in other organs like heart, lung, and kidney. These studies will also evaluate the persistence of high HbF expression over time, a parameter that is particularly critical for SCD patients, as levels below 20% are not considered protective. HSPCs constantly replenish all blood cell types through repeated cell divisions and differentiation steps. By editing HSPCs, the DNA modifications will be inherited by all the HSPC-derived blood lineages. DNA damage and DNA DSBs can lead to genotoxic effects [13,14,15,16] and loss of self-renewal in HSPCs. Moreover, unintended modifications can occur when similar DNA sequences are targeted, leading to the generation of off-target mutations that can potentially have deleterious consequences or provide selective advantages to specific blood lineages. The progressive transition to DSB-free CRISPR systems such as base and prime editing and the development of high-fidelity Cas enzymes with lower off-target activity [203] will help reduce the mutational risk. So far, no evidence of DSB-associated genotoxicity has been observed in patients receiving Casgevy. It might still be too early to rule out long-term genotoxicity, as two patients who underwent gene therapy for SCD developed blood malignancies 3–5.5 years post-transplant [204]. Clonal expansion has been observed in the patient who participated in the Graphite Bio trial; however, the clinical significance of this event remains unclear. Developing ways to reduce, detect, and predict genotoxicity of CRISPR-based therapies remains essential to safe clinical implementation. Most CRISPR-based therapies for genetic blood disorder require HSPC mobilization and myeloablation, a procedure associated with serious complications. Ex vivo genetic modification and transplantation of HSPCs is costly and can only be performed by specialized treatment centers with highly trained personnel, allowing a small portion of eligible patients to be treated each year. Moreover, since Casgevy could cost as much as USD 2 million per treatment, it would be practically inaccessible to low-income countries where the majority of TDT and SCD patients live [205].

Neuromuscular disorders cannot be corrected ex vivo and therefore rely on systemic viral delivery of the editing machinery, raising immunogenicity concerns both for the high AAV doses required and the long-term expression of CRISPR components [166]. Despite high transduction efficiency, durable therapeutic benefits, and remarkable clinical successes, the AAV platform suffers from several limitations. First, the limited cargo capacity limits the delivery options of CRISPR/Cas9 components, too large to be expressed from a single AAV vector. While dual-AAV systems are often used, all-in-one constructs [171] with truncated Cas9 nickase represent attractive solutions that are currently under investigation in vivo. The high dose required for therapeutic use has been previously associated with immunotoxicity in AAV-treated patients [200], and is a hurdle to manufacturing. Pre-existing immunity to AAVs and CRISPR components might limit access to therapies and will require careful monitoring and intervention pre- and post-infusion [206]. Optimized forms of Cas9 with modified epitopes hold great promise in reducing host immunogenicity [207]. In addition, the development of innovative AAV serotypes offering greater efficiency at lower doses [205] may overcome, in part, these challenges. Non-viral solutions such as gold and lipid nanoparticles that deliver mRNA to HSPC [203] and other tissues in vivo [204] may also play an important role in delivering CRISPR/Cas systems in the future. Alongside all these considerations, the durability of CRISPR/Cas9 treatment is pivotal for DMD and other muscle disorders. The efficient delivery of CRISPR/Cas9 elements to satellite cells may ensure long-lasting therapeutic benefits in growing patients. Whereas preclinical studies showed that systemic AAV CRISPR delivery can target satellite cells and editing persist up to 18 months, efficiency needs to be improved and further optimization is required to target this cell population.

The last decade witnessed the great potential of CRISPR/Cas9 systems, however extensive preclinical and clinical investigations are still needed to overcome several therapeutic challenges. Lessons learned from these studies and future CRISPR-based research in the hematological and neuromuscular fields will further the development of novel therapies for other devastating genetic diseases.

## Figures and Tables

**Table 1:** Clinical trials of blood diseases using CRISPR/Cas9 gene editing system.

Disease	Clinical trial	Therapeutic approach	Editor	Name	Sponsor	Study Phase
**SCD**	NCT03653247	Gene disruption of BCL11A using ZFN	ZFN	BIVV003	Sangamo Therapeutic (Richmond, CA, United States)	Phase 1/2
	NCT05329649	Gene disruption of BCL11A using CRISPR-Cas9	CRISPR-Cas9	CTX001	Vertex Pharmaceuticals Inc. (Boston, MA, United States)	Approved
	NCT03745287			Exa-cel		
	NCT05951205					
	NCT04443907	Gene disruption of BCL11A using CRISPR-Cas9	CRISPR-Cas9	OTQ923	Novartis pharmaceuticals (Basel, Switzerland)	Phase 1
	NCT05456880	Gene disruption of γ-globin promoter using adenine base editor	ABE	BEAM-101	Beam Therapeutics Inc. (Cambridge, MA, United States)	Phase 1/2
	NCT04853576	Gene disruption of γ-globin promoter using CRISPR-Cas12a	CRISPR-Cas12a	EDIT-301	Editas Medicine, Inc. (Cambridge, MA, United States)	Phase 1/2
	NCT04819841	Gene correction using CRISPR-Cas9 and AAV6	CRISPR-Cas9	CEDAR	Graphite Bio (San Francisco, CA, United States)	Phase 1/2
	NCT04774536	Gene correction using CRISPR-Cas9 and ssODN	CRISPR-Cas9	CRISPR_SCD001	Mark Walters, MD	Phase 1/2
**β-thalassemia**	NCT03432364	Gene disruption of γ-globin promoter using ZFN	ZFN	ST-400	Sangamo Therapeutics (Richmond, CA, United States)	Phase 1/2
	NCT05356195	Gene disruption of BCL11A using CRISPR-Cas9	CRISPR-Cas9	CTX001	Vertex Pharmaceuticals Inc. (Boston, MA, United States)	Approved
	NCT03655678					
	NCT04211480	Gene disruption of BCL11A using CRISPR-Cas9	CRISPR-Cas9	BRL-101	Bioray Laboratories (Boston, MA, United States)	Phase 1
	NCT05577312					
	NCT04390971	Gene disruption of γ-globin promoter using CRISPR-Cas9	CRISPR-Cas9	ET-01	EdiGene Inc. (Guangzhou, China)	Phase 1
	NCT05752123					
	NCT04925206					
	ChiCTR2100052858	Gene disruption of γ-globin promoter using CRISPR-Cas9	CRISPR-Cas9	RM-001	Guangzhou Reforgene Medicine (Guangzhou, China)	Phase 1
	ChiCTR2100053406					
	NCT05444894	Gene disruption of γ-globin promoter using CRISPR-Cas12a	CRISPR-Cas12a	EDIT-301	Editas Medicine, Inc. (Cambridge, MA, United States)	Phase 1/2
	NCT06041620	Gene disruption of γ-globin promoter using CRISPR-Cas12b	CRISPR-Cas12b	VGB-Ex01	Institute of Hematology & Blood Diseases Hospital (Tianjin, China)	Interventional
	NCT05442346	Gene disruption of γ-globin promoter using glycosylase base editors	Glycosylase base editors	-	Bioray Laboratories (Shanghai, China)	Phase 1/2
	NCT04205435	Gene correction of CVS-654 mutation using CRISPR-Cas9	CRISPR-Cas9	-	Bioray Laboratories (Shanghai, China)	Phase 1/2 Completed
	NCT03728322	Gene correction in induced HSCs using CRISPR-Cas9	CRISPR-Cas9	-	Allife Medical Science and Technology Co., Ltd. (Beijing, China)	Early phase 1
